# Large-Scale Expansion of Porcine Adipose-Derived Stem Cells Based on Microcarriers System for Cultured Meat Production

**DOI:** 10.3390/foods11213364

**Published:** 2022-10-26

**Authors:** Wenjuan Song, Peipei Liu, Huixia Li, Shijie Ding

**Affiliations:** 1 College of Animal Science and Technology, Nanjing Agricultural University, Nanjing 210095, China; 2 College of Food Science and Technology, Nanjing Agricultural University, National Center of Meat Quality and Safety Nanjing, Nanjing 210095, China

**Keywords:** adipose-derived stem cells, microcarrier, adipogenic differentiation, cultured fat

## Abstract

Cultured meat is an innovative meat-production technology that does not rely on animal husbandry. As a new food component, cultured fat is of great significance to cultured meat. In this study, we isolated adipose-derived stem cells (ADSCs) and identified the purity by immunofluorescence staining of ADSC-specific surface marker proteins CD44 and CD29 and showed that most of the cells were positive for CD29 and CD44. In addition, we detected the expression of *FABP4* and *Plin1* to confirm that ADSCs differentiated into mature adipocytes at 10 days post-induction. Subsequently, the culture conditions of ADSCs on microcarriers (MCs) were optimized and showed that cell density of living cells reached their highest after 5 days when continuously stirring at 50 rpm. Finally, the expression of *FABP4* and *PPARγ* was detected to confirm the adipogenic differentiation ability of ADSCs on 2D and 3D culture systems and showed that ADSCs maintained their adipogenic differentiation ability after expansion on MCs. In conclusion, this research demonstrated that reliance on MCs to expand ADSCs was a promising approach for production of cultured fat.

## 1. Introduction

With population growth and living standard improvements, global meat production and consumption are increasing [1,2]. It was reported that the demand for meat would increase 76% from 2005 by 2050. Increasing livestock production has led to a series of major environmental problems, including available freshwater consumption and greenhouse gas emissions [3,4], as well as animal welfare, and human health and food safety [5,6]. Therefore, finding a sustainable alternative production scheme is encouraged. Cultured meat is a novel form of meat production. It is edible muscle and adipose tissue produced by culturing animal cells in vitro using bio manufacturing according to the growth pattern of muscle or adipose tissue in the animal body [7]. In short, cultured meat is produced by culturing cells in the laboratory rather than by breeding animals. It has the potential to achieve sustainable large-scale meat production, thereby helping to address the environmental challenges associated with traditional livestock production [8]. 

So far, the research on cultured meat has mainly focused on cultured muscle tissue [9,10]. However, fat tissue has an important influence on the sensory and texture properties of meat [11]. For example, Japanese black cattle meat has increased juiciness and tenderness because of the high fat content [12]. In addition, it was reported that the content of intramuscular fat has a significant influence on flavor volatiles, which further indicates the contribution of fat to meat flavor and texture [13]. Thus, incorporation of cultured fat into cultured meat is very important for improving the quality of cultured meat.

Large-scale production of cultured fat in vitro requires seed cells, which can effectively differentiate into mature adipocytes after multiple population doublings. ADSCs, due to their relative ease of access and abundant and efficient differentiation into mature adipocytes, have been used to prepare cultured bovine adipose tissue in vitro [14,15]. In addition, a large number of seed cells is another challenge for the production of cultured fat. The expansion of ADSCs usually relies on a two-dimensional (2D) surface-adherent culture system, which is labor-intensive and cost-inefficient, and so it not suitable for preparing cultured meat [16]. However, MCs can ensure high-density cell cultures due to their larger surface-area-to-volume ratio, which provides a promising method for upscaling ADSC cultures [17]. Therefore, it is necessary to select a commercial MC and optimize the culture conditions of ADSCs on MCs to improve the scalability of ADSCs.

First, we isolated and identified porcine ADSCs. Second, the culture conditions, such as confirmation the time of terminal adipogenesis on 2D and expansion ADSCs on MCs were optimized. Finally, the adipogenic differentiation ability of ADSCs expanded by MCs was determined on a 2D surface and based on a 3D sodium alginate hydrogel system. Overall, this research aims to provide a promising seed cell candidate and a method for effective large-scale expansion of seed cells for promoting the development of cultured meat. 

## 2. Materials and Methods

### 2.1. ADSC Isolation and Culture

All animal experiments were approved by the Animal Welfare Committee of Nanjing Agricultural University and conducted in strict accordance with the guidelines and rules. The approval code is IACUC2020171. 

The adipose tissues were obtained from subcutaneous adipose tissue (SAT) of four male pigs (three days old). As previously described [18], fresh SAT was minced into 1–2 mm^3^ pieces and digested with collagenase (type I) (C0310, Sigma-Aldrich, Darmstadt, Germany) and terminated with growth medium (GM, DMEM/F12 with 10% FBS and 1% penicillin streptomycin). After digestion, the mixture was filtered with 100 μm cell strainers. Next, cells were centrifuged at 330× *g* for 5 min at room temperature (RT) and suspended in a red blood cell (RBC) lysis buffer for 5 min at RT. Cells were filtered with 40 μm cell strainers. Finally, cells were resuspended in GM and seeded into 10 cm cell culture plates with proliferation medium (supplemented with 5 μg/mL recombinant fibroblast growth factor 2 (FGF2) in GM) at a density of 2 × 10^3^ cells/cm^2^ in a 37 °C 5% carbon dioxide (CO_2_) incubator (ThermoFisher Scientifific, Waltham, MA, USA). After 12 h, the adherent cells were porcine ADSCs. The proliferation medium was changed every two days. For digestion, cells were detached with 1 mL of 0.25% trypsin (Sigma Aldrich, Castle Hill, Australia) for 1 min at 37 °C and neutralized with GM after being cultured for three days. The cell suspensions were collected in 15 mL plastic tubes and centrifugated for 5 min at 330× *g*. Finally, the pellets were suspended in the GM for experiments.

### 2.2. Expansion of ADSCs on MCs

MCs were added to a 150 mL spinner flask. Cells were seeded with a density of 4 × 10^4^ cells/mL into the spinner flask containing 120 mL proliferation medium at first 24 h with continuous stirring at 50 rpm or stirring for 5 min every 2 h. After 24 h, the stirring speed was maintained at 50 rpm. Half of the proliferation medium was replaced every 2 days and cultured for 6 days. Cell count was performed on an automated cell counter. For obtaining cells, MCs were digested with a special MC lysis buffer at 37 °C for 30 min until the MCs were completely lysed and centrifuged at 330× *g* for 5 min. Finally, the cells were resuspended in GM, and counted for subsequent experiments.

### 2.3. Live/Dead Viability Assay

The cell viability of ADSCs cultured on MCs was evaluated with a live/dead viability assay kit (KeyGen Biotech. Co., Ltd., Nanjing, China) on day 3, 4, 5, and 6. According to the manufacturer’s instructions, the ADSCs were incubated with a live/dead staining reagent in the dark at 37 °C for 30 min. Images were then taken by a fluorescence microscope, in which the live cells and dead cells respectively show as green and red.

### 2.4. Preparation of 3D Hydrogel

Two accurately-weighed grams of sodium alginate were sterilized with ultraviolet irradiation for 2 h and fully dissolved in 100 mL ultrapure water to prepare 2% sodium alginate solution. Then, one accurately weighed gram of calcium chloride (CaCl_2_) was dissolved in 100 mL of ultrapure water to prepare 1% CaCl_2_ solution and filtered by a 0.22 μm filter. ADSCs were gently resuspended in sodium alginate solution with a final density of 2 × 10^7^ cells/mL. Then, the sodium alginate solution containing ADSCs was uniformly covered on a 6-well plate. A quantity of 3 mL of CaCl_2_ was slowly added for cross-linking for 2 min and then rinsed with PBS to remove CaCl_2_ residue. The ADSCs were cultured with a proliferation medium on Day 1 and replaced the adipogenic differentiation medium (ADM) on Day 2. 

### 2.5. Adipogenic Differentiation

**Figure 1:**
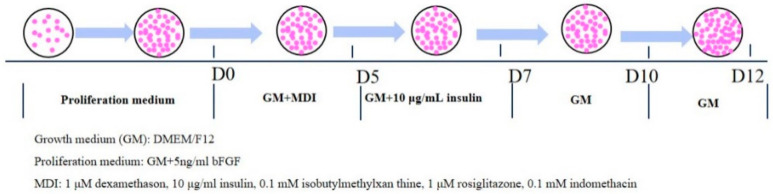
The time flow diagram for inducing ADSC to differentiate into mature adipocytes.

For 2D adipogenic differentiation, ADSCs with a density of 5 × 10^4^ per 3.5 cm cell culture dish were seeded (Corning Incorporated, NY, USA) with proliferation medium and changed the medium every two days until cell confluence. For 3D adipogenic differentiation, the proliferation medium of ADSCs in 3D hydrogel was replaced with the ADM on Day 2. Adipogenic differentiation of ADSCs was induced in a 37 °C 5% CO_2_ incubator. The time flow diagram for inducing ADSC to differentiate into mature adipocytes was shown in Figure 1.

### 2.6. Nile Red Staining

Lipid droplet visualization of ADSCs cultured in 2D was evaluated using Nile red staining. Briefly, the ADSCs were fixed with 4% paraformaldehyde at RT for 15 min. Cells were then washed three times with PBS and incubated with Nile red working solution for 15 min at RT. The cells were washed three times with PBS. Images were then taken using an inverted fluorescence microscope. 

### 2.7. Quantificational of Real-Time Polymerase Chain Reaction (qRT-PCR)

Total RNA was isolated by using an RNA extraction kit (Biochemical Technology (Beijing) Co., Ltd., Beijing, China) according to the manufacturer’s instruction and quantified by NanoDrop spectrophotometer. Total RNA was reverse-transcribed using a high-capacity cDNA synthesis kit (Nanjing Vazyme Biotechnology Co., Ltd., Nanjing, China) and then subjected to a real-time polymerase chain reaction (PCR) with SYBR Green master mix (Vazyme, China) on a Bio-Rad CFX. The sequences of forward and reverse primers were as follows: 5′-GTCGGAGTGAACGGATTTGGC-3′ and 5′-CTTGCCGTGGGTGGAATCAT-3′ for GAPDH; 5′-TGGCCATTCGCATCTTTCAG-3′ and 5′-ATCTCGTGGACGCCATACTT-3′ for *PPARγ*; 5′-AGAAGTGGGAGTGGGCTTTG-3′ and 5′-ATGATCAGGTTGGGTTTGGC-3′ for *FABP4*; 5′-GAGCCCGGCAACTCTAGTAT-3′ and 5′-CCCTACTCGGTAGGAATCGG-3′ for *C/EBPα*; 5′-CCGACCGAATGCAGAAGGA-3′ and 5′-ACAGAGTATTTGCGCTCCGGA-3′ for *C/EBPβ*.

5′-CAGTTCACAGCTGCCAATGA-3′ and 5′-TTCAGCTCAGAGGCGATCTT-3′ for *Plin1*. The internal normalization control was GAPDH. 

### 2.8. Western Blot

The 2D and 3D ADSCs were lysed with RIPA containing 1% phenyl methyl sulfonyl fluoride (PMSF). Protein concentration was determined in the samples using BCA (Beyotime Biotechnology, Shanghai, China). Then, the samples were detected by sulfate polyacrylamide gradient gel electrophoresis (SDS-PAGE) and transferred to PVDF membranes. The membranes were incubated with 5% BSA for 2 h, then incubated in the primary antibody (anti-FABP4, 2120s, CST; anti-PPARγ, 2443s, CST; anti-plin 1, ab61682, abcam; anti-GAPDH, ab2302, Merck) overnight. On the second day, the membrane was incubated in the secondary antibody for 2 h at RT and then washed with TBST 3 times. Finally, the bound antibodies were visualized with a chemiluminescence detection kit (ECL; Beyotime Biotechnology, Shanghai, China). The protein was quantified by density analysis with Image J FIJI. GAPDH was used as an internal control.

### 2.9. H&E Staining

The SAT of a pig was collected and fixed in 10% paraformaldehyde for 24 h, then embedded in paraffin, sectioned, and stained by hematoxylin and eosin (H&E), and imaged by bright field microscope.

### 2.10. Statistical Analysis

For qRT-PCR analysis, the data were shown as the fold change relative to the control. Statistical analysis was analyzed by Duncan’s multiple range method performed using SPSS 20. The data were presented as bar graphs with error bars representing standard deviation. The data were considered significantly different if *p* < 0.05. 

## 3. Results and Discussion

### 3.1. Structure and Vascular Distribution of Adipose Tissue in Porcine

**Figure 2:**
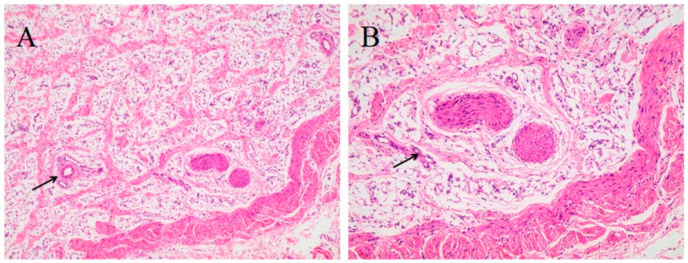
Morphological characteristics of piglet’s SAT by H&E staining. (**A**) The image magnified (10×). (**B**) The image magnified (20×). Arrows suggest SVF in SAT.

It is reported that ADSCs originate from perivascular cells [19,20]. Therefore, we first observed the distribution of blood vessels in adipose tissue by hematoxylin and eosin (H&E) staining. As shown in Figure 2A,B, a large number of vascular-like structures were observed in the SAT, including both large blood vessels in the lumen and small blood vessel branches from large blood vessels. The vascular wall presents a three-layer arteriovenous-like structure and capillary-like monolayer structures. This structural region was termed the stromal vascular fraction (SVF) [21]. Furthermore, a large number of single-chamber lipid droplets could be seen in the adipose tissue owing to the high magnification. The lipid droplets were dissolved into the vacuoles and the nucleus was pushed to the edge of the cell by the lipid droplets. There was a thin layer of connective tissue between the cells, and there may be invisible microcapillaries. 

### 3.2. Isolation and Identification of Porcine ADSCs

**Figure 3:**
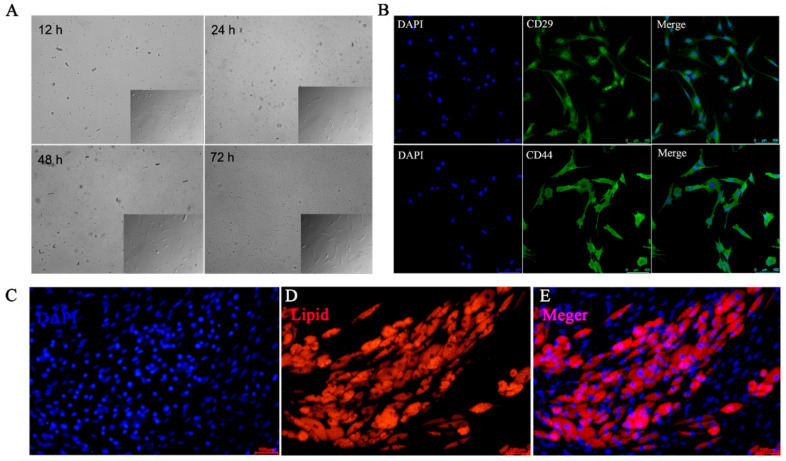
Isolation and identification of porcine ADSCs. (**A**) Cell morphology of ADSCs after isolation for 12–72 h. (**B**) Immunofluorescence staining of ADSCs surface-marker proteins CD29 and CD44. (**C**) 4′,6-diamidino-2-phenylindole (DAPI) staining of ADSCs induced by ADM. (**D**) Nile red staining for lipids. (**E**) The merged images of DAPI and Nile red.

Having determined that there was a subpopulation of cells in SAT by morphological staining, we next performed collagenase digestion to isolate SVF according to previous reports. The SVF consisted of a heterogeneous cell population, including ADSCs and hematopoietic precursors, mature vascular endothelial and progenitor cells, pericytes, fibroblasts, granulocytes, monocyte/macrophages and lymphocytes [22,23]. Subsequently, according to the characteristics that ADSCs adhere faster than precursor adipocytes and endothelial cells, the nonadherent cells were removed after 12 h, thus ADSCs were effectively purified by changing the medium. The cells appeared fibroblast-like with scattered adherent and the cells without adhesion were spherical or round at the first 12 h (Figure 3A). After 48–72 h, the number of adherent cells increased rapidly, and the cell’s morphology were a typical spindle [24,25] (Figure 3A). Then, CD29 and CD44, well-known specific surface-marker proteins of ADSCs, were used to identify the cells of passage two by immunofluorescence staining. The results showed that most of the isolated cells were positive for CD44 and CD29 [25] (Figure 3B). In addition, the simplicity and efficiency of adipogenic differentiation are crucial to evaluating the applicability of ADSCs for cultured fat production [26]. In general, differentiation of ADSCs into mature adipocytes is a simple process, which uses a four-component hormone cocktail of insulin, dexamethasone, isobutylmethylxanthine, and indomethacin. In this research, rosiglitazone, a PPARγ agonist, was also added to the cocktail to promote the ADSCs to differentiate into mature adipocytes [27]. Here, in order to detect the adipogenic differentiation ability of ADSCs, P5 ADSCs were induced by adipogenic differentiation with ADM and lipids visualized by Nile red staining. The result showed that ADSCs could effectively accumulate lipids (Figure 3C–E). Collectively, it was indicated that high-purity ADSCs were isolated and worthy of further research as candidate cells for cultured fat. 

### 3.3. Confirmation the Terminal Adipogenesis of ADSCs

**Figure 4:**
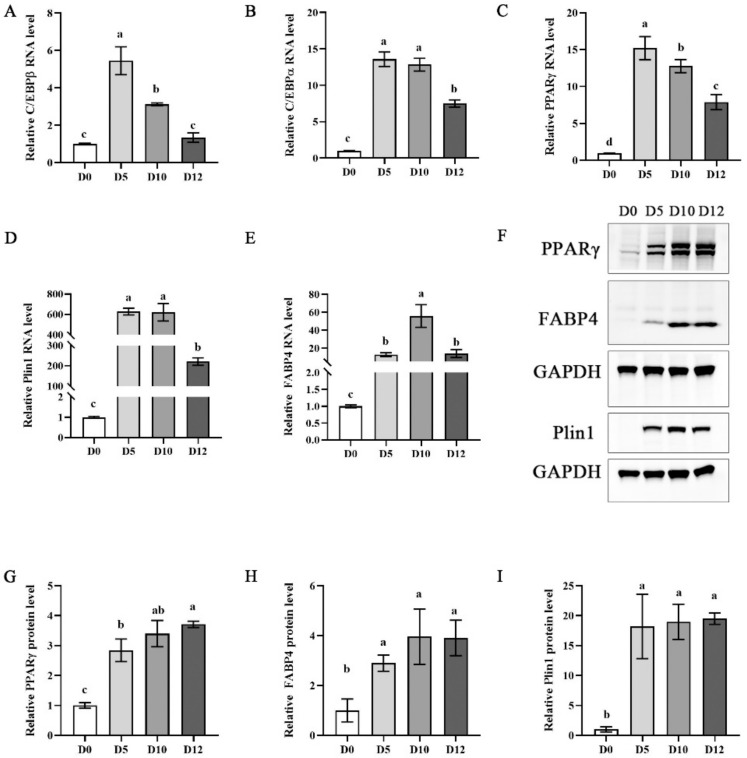
Confirmation the terminal adipogenesis of ADSC. (**A**–**E**) qPCR analysis the expression of adipogenic differentiation marker genes *C/EBPβ*, *C/EBPα*, *PPARγ*, *FABP4* and *Plin1*. (**F**) Western blot analysis the expression of adipogenic differentiation marker proteins *PPARγ*, *Plin1* and *FABP4*. (**G**–**I**) Quantitative adipogenic differentiation marker proteins *PPARγ*, *Plin1* and *FABP4*. Statistical testing: a–d, different letters in the same columns indicate significant differences. *p* < 0.05, and error bars indicate SEM.

The robust differentiation ability of ADSCs is another vital requirement for the preparation of cultured fat. It is crucial that the terminal adipogenesis of ADSCs for cultured meat is confirmed. To define the sequence of events leading to terminal adipogenesis of ADSCs, we performed qPCR to detect the expression of marker genes at different stages of differentiation. As shown in Figure 4A–C, the genes of *C/EBPβ*, *C/EBPα* and *PPARγ* were relatively higher on day 5. *C/EBPβ* and *C/EBPδ* simultaneously control expression of both *PPARγ* and *C/EBPα* [28]. It was reported that *PPARγ* was the master regulator of adipogenesis [29] and regulated adipogenesis by regulating the expression of the adipose-specific fatty acid binding protein aP2/FABP4 [30]. Subsequently, the expressions of *Plin1* and *FABP4* were relatively higher on day10, but the expression of these genes decreased on day 12 (Figure 4D,E). The role of *FABP4* is responsible for the transport of fatty acids to the cell membrane and is a marker of mature adipogenesis [31]. *Plin1*, a lipid-droplet surface protein, provides a host site for lipid storage and release, and responses for stabilizing lipid maturation marker vesicles [32,33]. We further detected the expression of key adipogenic proteins *Plin1*, *FABP4* and *PPARγ* in the process of differentiation. The results showed that *Plin1* and *FABP4* were not expressed on day 0 of differentiation, the expression of *Plin1* and *FABP4* increased gradually with the extension of differentiation, the expression level was greatest on day 10, and there was no significant difference on day 12. However, the expression of *PPARγ* reached its highest on day 5 of differentiation (Figure 4F–I). Taken together, these results indicated that terminal fat was formed on day 10 of inducing adipogenic differentiation of ADSC.

### 3.4. Large-Scale Expansion of ADSCs Based on MCs

**Figure 5:**
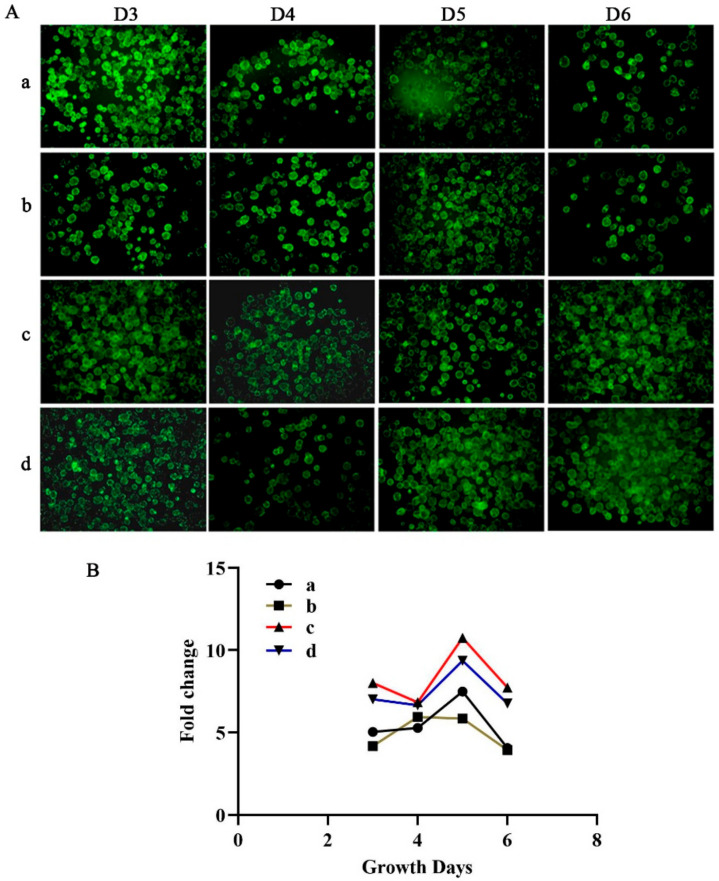
Large scale expansion of ADSCs based on MCs. (**A**) Evaluated the occupancy and confluency of live ADSCs on MCs stained by live/dead staining. (**B**) Fold change of ADSCs cultured on MCs.

Large-scale expansion of ADSCs is one of the requisites for cultured fat [10]. In order to increase the scale expansion of ADSCs, it is necessary to improve the efficiency of the number of cells cultured in each unit of medium [34]. The MCs can provide a larger surface area per unit volume of medium; thus, the stirring culture system based on MCs has been widely used in large-scale expansion cells [35]. In this research, ADSCs were seeded on MCs in a rotating flask system and optimized the expansion efficiency of ADSCs by optimizing the stirring speed, culture time, and culture volume (Table 1). The adhesion efficiency of ADSCs on MCs reached 97.7% on day 1. Following this, we evaluated cell proliferation on MCs by live/dead staining. The results showed that occupancy and confluency of live cells (green) visibly increased on MCs on day 5, compared with that at Day 3 and 6 under the four culture conditions (Figure 5A). Moreover, the fold changes of ADSCs were detected on days 3, 4, 5, and 6. The results showed that after 5 days of culture, the number of ADSCs on MCs reached the highest in group a-d, which were 7.49, 5.86, 10.76, and 9.36 folds, respectively (Figure 5B). In addition, ADSCs were inoculated on MCs within 24 h, more cells were obtained by continuously stirring at 50 rpm than stirring for 5 min every 2 h after 5 days of culture (Figure 5B), and the number of cells of the working volume of 90 mL was more than that of 60 mL for 5 days of culture (Figure 5B). Subsequently, we expanded the working volume of the MCs to 500 mL, cell density increased 5–7-fold with continuous stirring at 50 rpm during 5 days of culture. The density of cells on MCs did not reach 26-fold as described by Dohmen et al. [36]. Thereby, more research should be carried out to select the appropriate MCs (different surface chemistry, material, size, and porosity) [37], optimizing culture conditions (stirring speed, culture time) and other factors to enable ADSCs of piglets to expand more effectively in vitro in the future. In conclusion, these results show that ADSCs could be efficiently expanded in MCs-based systems.

### 3.5. Validation of Adipogenic Differentiation Ability of ADSCs after Expansion on MCs

**Figure 6:**
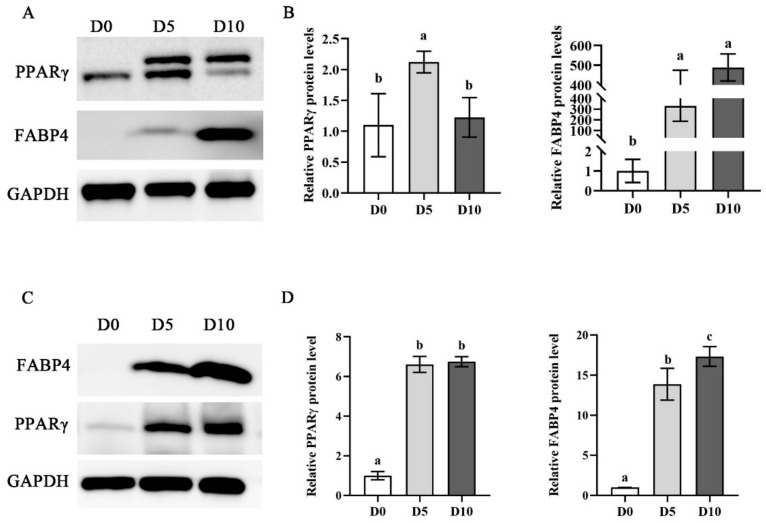
Validation of adipogenic differentiation ability of ADSCs after expansion on MCs. (**A**) Western blot analysis of the expression of adipogenic differentiation of marker proteins *PPARγ* and *FABP4* for 2D surface culture. (**B**) Quantitative adipogenic differentiation of marker proteins *PPARγ* and *FABP4* for 2D surface culture. (**C**) Western blot analysis of the expression of adipogenic differentiation of marker proteins *PPARγ* and *FABP4* for 3D culture. (**D**) Quantitative adipogenic differentiation of marker proteins *PPARγ* and *FABP4* for 3D culture. Statistical testing: a–c, different letters in the same columns indicate significant differences. *p* < 0.05, and error bars indicate SEM.

The adipogenic differentiation ability of ADSCs after large-scale expansion is a prerequisite for preparing cultured fat, it is thus necessary to confirm the adipogenic differentiation ability of ADSCs after being expanded on MCs. Here, we seeded ADSCs on the 2D surface and induced adipogenic differentiation when the cell density reached more than 90%. The expression of *PPARγ* and *FABP4* proteins was detected by Western blot. The results showed that the expression of *PPARγ* increased 2.12 ± 0.18-fold on day 5 of adipogenic differentiation, and the expression of *FABP4* increased y 489.3 ± 68.8-fold on day 10 of adipogenic differentiation, compared with Day 0 (Figure 6A,B). In addition, we examined the adipogenic differentiation ability of ADSCs in 3D hydrogels. As previously described, ADSCs were encapsulated in sodium alginate hydrogel and induced to differentiate into mature adipocytes and detected the expression of *PPARγ* and *FABP4* proteins by Western blot. As shown in Figure 6C,D, the results showed that the expression of *FABP4*, a marker protein of mature adipocytes, highly up-regulated at day 10 of adipogenic differentiation. The expression of *PPARγ* showed that its expression was significantly higher on day 5, compared with before induction (Figure 6C,D). Taken together, our results demonstrated that ADSCs are a promising candidate cell type for the production of cultured fat.

## 4. Conclusions

In this research, we isolated high-purity ADSCs from pSAT. Next, ADSCs were differentiated into mature adipocytes on day 10 in the adipogenic cocktail of our laboratory. In addition, ADSCs proliferated 5–7-fold after the culture spent 5 days on MCs with continuous stirring at 50 rpm, and still maintained the capacity of adipogenic differentiation. Overall, this study provides a promising candidate cell type for the production of cultured fat in future and proposes a prospective method for the expansion ADSCs based on MCs.

## Figures and Tables

**Table 1:** Optimization of the culture conditions of ADSCs on MCs.

Groups	Working Volume (mL)	Mixing Conditions		Feeding
		0–24 h	After 24 h	
a	60	50 rpm	50 rpm	50%/D3; 25%/D4&25%/D5
b	60	50 rpm, 5 min; 0 rpm, 2 h	50 rpm	
c	90	50 rpm	50 rpm	
d	90	50 rpm, 5 min; 0 rpm, 2 h	50 rpm	

## Data Availability

All the data of this research are included in this manuscript.
